# Incidence of bovine clinical mastitis in Jammu region and antibiogram of isolated pathogens

**DOI:** 10.14202/vetworld.2017.984-989

**Published:** 2017-08-25

**Authors:** Adil Majid Bhat, Jasvinder Singh Soodan, Rajiv Singh, Ishfaq Ahmad Dhobi, Tufail Hussain, Mohammad Yousuf Dar, Muheet Mir

**Affiliations:** 1 Division of Clinical Veterinary Medicine, Faculty of Veterinary Sciences and Animal Husbandry, Sher-e-Kashmir University of Agricultural Sciences & Technology of Kashmir, Shuhama, Ganderbal, Jammu and Kashmir, India; 2 Teaching Veterinary Clinical Complex, Faculty of Veterinary Sciences and Animal Husbandry, Sher-e-Kashmir University of Agricultural Sciences and Technology of Jammu, R.S. Pura, Jammu, Jammu and Kashmir, India; 3 Division of Veterinary Medicine, Faculty of Veterinary Sciences and Animal Husbandry, Sher-e-Kashmir University of Agricultural Sciences and Technology of Jammu, R.S. Pura, Jammu, Jammu and Kashmir, India; 4 Division of Animal Nutrition, Faculty of Veterinary Sciences and Animal Husbandry, Sher-e-Kashmir University of Agricultural Sciences and Technology of Jammu, R.S. Pura, Jammu, Jammu and Kashmir, India; 5 Division of Veterinary Anatomy, Faculty of Veterinary Sciences and Animal Husbandry, Sher-e-Kashmir University of Agricultural Sciences and Technology of Jammu, R.S. Pura, Jammu, Jammu and Kashmir, India

**Keywords:** antimicrobial sensitivity, bovines, clinical mastitis, incidence

## Abstract

**Aim:**

This study was conducted to evaluate the incidence of clinical mastitis in bovines of Jammu region, to identify the infectious organisms responsible for it, and the antimicrobial sensitivity of isolated pathogens.

**Materials and Methods:**

The study was conducted on cases that were presented to the Medicine Division of Teaching Veterinary Clinical Complex, Faculty of Veterinary Sciences and Animal Husbandry, R.S. Pura, Jammu, Jammu and Kashmir. A total of 260 cases of bovines were presented from June 30, 2012, to July 01, 2013, out of which 30 cases were of clinical mastitis. The diagnosis of clinical mastitis was made on the basis of history and clinical examination of affected animals.

**Results:**

Animal and quarter-wise incidence of clinical mastitis were found to be 11.5% and 5.76%, respectively. Of the 23 isolates obtained, *Staphylococcus aureus* (60.87%) was the most frequently isolated organism, followed by coagulase negative *Staphylococci* (13.04%), *Streptococcus uberis* (4.35%), *Streptococcus dysgalactiae* (8.69%), and *Escherichia coli* (13.04%). The antimicrobial sensitivity of isolates revealed maximum sensitivity to enrofloxacin, gentamicin, amoxicillin/sulbactam, ceftriaxone/tazobactam, ceftizoxime, ampicillin/sulbactam and least sensitivity for oxytetracycline and penicillin.

**Conclusion:**

*Staphylococcus* spp. is the major causative agent of clinical mastitis in bovines of Jammu region. The causative agents of the clinical mastitis were most sensitive to enrofloxacin and gentamicin.

## Introduction

Clinical mastitis is the frequently occurring and economically important disease for dairy industries worldwide [1]. The incidence of clinical mastitis is an important indicator of animal health and welfare. The decrease in incidence of clinical mastitis has a positive effect on animal health, animal welfare, antimicrobial use, work pleasure, and net return of the farm [2]. The incidence of clinical mastitis is associated with many risk factors, and the sampling unit in risk factor studies can vary from quarter level to herd level. Quarter-specific risk factors are responsible for the difference in clinical mastitis occurrence in different quarters of the same animal. Cow-specific risk factors are related to the difference in clinical mastitis incidence among cows. Parity, month of lactation, season of the year, somatic cell count in previous lactation, and clinical mastitis history are the cow-specific risk factors that are currently known [3]. A wide variety of microorganisms including bacteria, fungi, yeast, and mycoplasma are responsible for causing mastitis, of which bacteria are the most frequently isolated pathogens. The mastitis causing pathogens can be classified as contagious and environmental pathogens. The major contagious pathogens comprise *Streptococcus agalactiae, Staphylococcus aureus*, and *Mycoplasma bovis*, and the major environmental pathogens include *Enterobacteriaceae* (particularly *Escherichia coli*) and *Streptococcus uberis*. Coagulase negative *Staphylococci* infections tend to be subclinical and coliform infections tend to be clinical [4].

In India, annual economic losses due to subclinical and clinical mastitis have been estimated to be Rs. 41.511 and Rs. 30.144 billions, respectively, with a total of Rs. 71.655 billions [5]. The losses caused by clinical mastitis arise mostly from costs of treatment, culling of animals, death, and decreased milk production. Apart from financial losses, the importance of mastitis with regard to public health should not be overlooked. The extensive use of antibiotics in treatment and control of mastitis has possible implications on human health, through emergence of antibiotic resistant bacteria [6].

The objectives of our study were to study the incidence of clinical mastitis in bovines of Jammu region and antibiogram of organisms isolated from clinical cases. The antibiotic sensitivity profile of isolated pathogens can serve as a guide for field veterinary practitioners to provide effective and timely treatment to animals affected with clinical mastitis.

## Materials and Methods

### Ethical approval

This study was conducted keeping all ethical and animal welfare issues under consideration and it was approved by Institutional animal ethics committee, registered by CPCSEA, under registration number P62/ac/04/cpcsea dated: 16/12/2004.

### Selection of animals

A total of 260 cases of bovines, from June 30, 2012, to July 01, 2013, were presented to the Medicine Division of Teaching Veterinary Clinical Complex, Faculty of Veterinary Sciences and Animal Husbandry, R.S. Pura, Jammu, out of which 30 cases were of clinical mastitis. The detailed history of animals was taken from owners, including age, parity, date of calving, duration of illness, and milk yield. The general clinical examination was done, which was followed by specific examination of udder. The affected quarters were hot, swollen, and painful on touch.

### Milk quality

The milk from most cases of clinical mastitis showed the presence of flakes and pus. No animals had blood stained milk from affected quarters.

### Method of milking

All animals were hand milked.

### Parity of animals

The number of animals that belonged to first, second, third, fourth, fifth, sixth, and above parity was 4, 7, 3, 9, 6, and 1, respectively.

### Collection of milk samples

Milk samples from individual quarters of animals were taken after washing the udder with antiseptic solution (Mastinil solution by natural remedies) for visible debris, and teat ends were scrubbed with cotton soaked in spirit. The first three or four streaks of milk were discarded, and next 15-20 ml of milk was collected in autoclaved vials for culture and sensitivity test.

### Bacteriological examination

**Figure-1:**
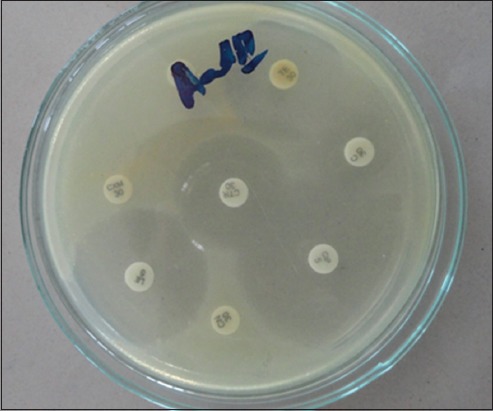
Antibiotic sensitivity test of isolates on Mueller Hinton agar.

**Figure-2:**
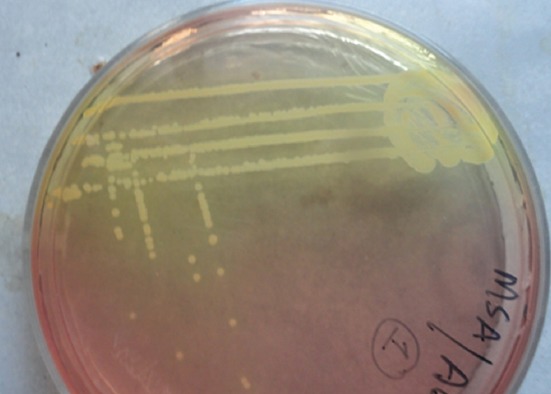
Growth of *Staphylococcus aureus* on Mannitol salt agar.

**Figure-3:**
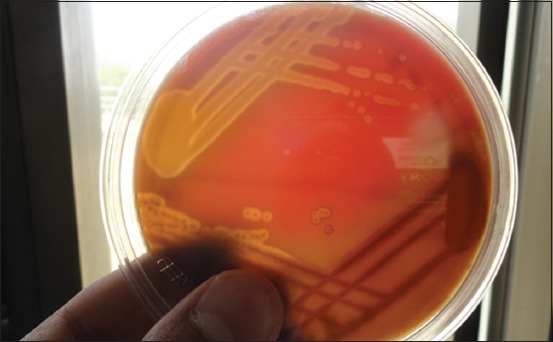
Growth of *Streptococci* on Edwards media showing esculin hydrolysis.

**Figure-4:**
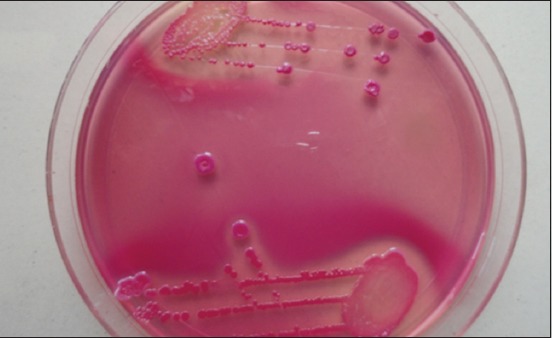
Growth of *Escherichia coli* on MacConkey agar.

The isolation of microorganisms was done as per Quinn *et al*. [7]. Milk samples collected in sterile glass vials were streaked primarily on ovine blood agar plates with a sterile platinum loop under strict sterile environment. The inoculated plates were incubated at 37°C for 24 h. The causative organisms were identified initially by colony characteristics on blood agar, Gram-staining and biochemical characteristics for the presence of catalase and cytochrome C oxidase. Further, the organisms grown on blood agar plates were streaked on their selective media. Mannitol salt agar was used to grow *Staphylococcus* spp. (Figure-1), Edward’s media for *Streptococci* (Figure-2), MacConkey agar and eosin methylene blue agar for coliforms and *Enterococci* (Figures-3 and 4, respectively). Christie–Atkins–Munch-Petersen (CAMP) test and esculin hydrolysis on Edwards media were used for species identification of *Streptococci*.

### Antibiotic sensitivity test

**Figure-5:**
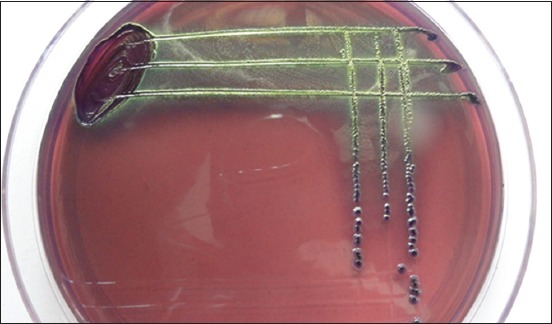
Growth of *Escherichia coli* on eosin methylene blue agar.

Antibiotic sensitivity test of individual bacterial isolates to standard antibiotics was determined by disc diffusion method [8]. About 3-4 colonies of the pathogenic organisms grown on blood agar were dipped in nutrient broth under sterile condition and kept for 2 h for the appearance of turbidity. The culture from nutrient broth was poured on to Mueller-Hinton medium plate and spread evenly (Figure-5). Then, the plates were incubated for 24 h, and the zone of inhibition for each antibiotic was noted and compared with standard table. A total of 14 antibiotic discs were tested against different microbial isolates including, oxytetracycline (30 µg), enrofloxacin (10 µg), gentamicin (10 µg), penicillin (2 µg), ceftriaxone (30 µg), cefotaxime (30 µg), ceftriaxone/tazobactam (20/10 µg), amoxicillin/clavulanic acid (20/10 µg), cefoperazone (30 µg), ampicillin/sulbactam (20/10 µg), neomycin (30 µg), ciprofloxacin (1 µg), ceftizoxime (30 µg), and tylosin (30 µg).

## Results and Discussion

The cow wise and quarter wise incidence of clinical mastitis were found to be 11.5% and 5.76%, respectively. Our results were in agreement with other workers such as Ghose *et al*. [9] and Tolosa *et al*. [10], who reported the prevalence of 4.06% and 4.77%, respectively. Occurrences of clinical mastitis varying from 3.77% to 23% have been reported by Riekerink *et al*. [11] and Sharma *et al*. [12]. The incidence was highest in animals falling in fourth parity (30%) followed by those in third and fifth parity (Table 1). Verbeke *et al*. [13] have reported higher incidence rate of clinical mastitis in higher parity cows. The increased incidence of mastitis with parity may be ascribed to loosening of sphincter and patency of teat canal in older cows. Moreover, the median ligaments which provide support to the teat also get relaxed with age leading to hanging of udder and thus making it more prone to mastitis. The incidence was also influenced by milk yield of animals. The incidence of clinical mastitis was highest in animals having milk yield between 10 and 20 L (36.67%). Our findings were in agreement with works done by Sudhan *et al*. [14] and Tiwari *et al*. [15]. The high milk yield may lead to loss of micronutrients that have role in immunity. Based on species of animals affected, incidence was highest in cattle (73.33%) than buffaloes (26.67%). This lower prevalence of mastitis in buffaloes can be due to strong smooth muscles sphincter around the teat opening, which reduces their susceptibility to intramammary infection (Table 1) [16]. All animals were hand milked, and the relationship between hand milking and clinical mastitis may be attributed to faulty milking procedures. The knuckling method is most commonly practiced in rural areas that may cause injury to teat cistern and predispose to mastitis The presence of teat injuries or lesions were found to be a risk factor for clinical mastitis, intramammary infection (IMI) any pathogen and *Staphylococcus* spp. IMI [17]. The teat skin forms the first line of defense against mastitis pathogens and loses its protective characteristics when injured, facilitating bacterial colonization [18]. The bacteriological examination of milk from animals suffering from mastitis revealed a total of 23 bacterial isolates. Among the recovered bacterial isolates, *S. aureus* had 14 isolates (60.87%), *Streptococcus dysgalactiae* two isolates (8.69%), coagulase negative *Staphylococci* three isolates (13.04%), *S. uberis* one isolate (4.35%), and coliform three isolates (9.09%). High prevalence of *Staphylococcus* spp. observed in this study was in agreement with findings of Kurjogi and Kaliwal [19], Ranjan *et al*. [20], Keane *et al*. [21], and Verbeke *et al*. [13]. The reason for the predominance of contagious pathogens, particularly *Staphylococcus* spp. may be ascribed to the ubiquitous nature of this organism and drug resistance shown by them. Further, a total lack of contagious mastitis pathogens control practices as post-milking antiseptic teat dipping, dry period antibiotic therapy, culling of chronically infected animals in the herd and rife proclivity of using milk foam in the milking pail to lubricate the teat during milking, could also be reason for their high prevalence (Table 2). The bacterial isolates of *Staphylococci* spp., *Streptococcus sp*., and *Escherichia coli* were highly sensitive to enrofloxacin and gentamicin, followed by amoxicillin/clavulanic, ampicillin/sulbactam, ceftriaxone/tazobactam, and tylosin. Efficacy of oxytetracycline, ceftriaxone, cefotaxime, cefoperazone, ciprofloxacin, and ceftizoxime was moderate. Penicillin was the least effective antibiotic, with no isolate showing susceptibility (Table 3). High sensitivity to enrofloxacin and gentamicin has also been reported in previous studies [20,22-24]. Similar results were reported by Kaliwal *et al*. [25] and Awadkar and Kulkarni [26]. Moderate resistance shown by oxytetracycline in the present study is comparable with that reported in Finland [26], while in contrast, Alekish *et al*. [27] and Mahami *et al*. [28] reported 100% resistance to tetracyclines. The rate of penicillin resistance (100%) observed in this study is much higher than those reported by other workers, such as Pitkala *et al*. [29], Rajala-Schultz *et al*. [30], Kenar *et al*. [31]. Resistance to penicillin in *Staphylococci* has been associated with the production of β-lactamases and low-affinity penicillin-binding protein [32]. Besides, the high resistance among bacterial isolates to penicillin could be attributed to indiscriminate use of antibiotics in clinical cases without following proper dosage regimen.

**Table-1:** Incidence of clinical mastitis in relation to various risk factors.

Risk factor	Groups	Quarters affected	Total quarters	Percent incidence
Position of quarter	Left fore	14	60	23.33
	Right fore	10		16.67
	Right hind	19		31.67
	Left hind	17		28.33

**Risk factor**	**Groups**	**Animals affected**	**Total animals**	**Percent incidence**

Parity of animals	First parity	4	30	13.33
	Second	7		23.33
	Third	3		10.0
	Fourth	9		30.0
	Fifth	6		20.0
	Sixth and above	1		3.34
Milk yield	0-10 L	11	30	36.67
	10-20 L	17		56.67
	>20 L	2		6.66
Age in years	3-5	14	30	46.67
	5-8	12		40
	>8	4		13.33

**Table-2:** Organisms isolated from milk samples of bovines suffering from clinical mastitis.

Organisms isolated	Number of isolates n (%)
*Staphylococcus aureus*	14 (60.87)
Coagulase negative *Staphylococci*	3 (13.04)
*Streptococcus uberis*	1 (4.35)
*Streptococcus dysgalactiae*	2 (8.69)
*Escherichia coli*	3 (13.04)
Total	23 (100)

**Table-3:** Sensitivity pattern of microorganisms isolated from clinical mastitis cases to various antimicrobial agents.

Antibiotic	Bacterial isolates								

	*Staphylococci*			*Streptococci*			*Escherichia coli*		
		
	S	I	R	S	I	R	S	I	R
Oxytetracycline	10	2	5	1	0	2	1	0	2
Enrofloxacin	16	1	0	3	0	0	3	0	0
Gentamicin	16	0	1	3	0	0	3	0	0
Penicillin	0	0	17	0	0	3	0	0	3
Ceftriaxone	11	1	5	2	1	0	1	0	2
Cefotaxime	9	7	1	2	1	0	1	0	2
Ceftriaxone/tazobactam	14	3	0	3	0	0	3	0	0
Amoxicillin/clavulanic	15	2	0	3	0	0	2	0	1
Ampicillin/sulbactam	15	2	0	3	0	0	2	1	0
Cefoperazone	11	3	3	2	1	0	2	0	1
Neomycin	12	3	2	2	1	0	2	0	1
Ciprofloxacin	12	5	0	2	1	0	2	0	1
Ceftizoxime	12	4	1	2	1	0	1	0	2
Tylosin	14	2	1	2	1	0	1	0	2
Total	17			3			3		

## Conclusion

The study concluded that *Staphylococcus* spp. is the major etiological agent of clinical mastitis in bovines of Jammu region. Based on antibiotic sensitivity test, enrofloxacin and gentamicin were found to be most sensitive antibiotics against microorganisms causing clinical mastitis. There is an urgent need to enhance awareness among the dairy farmers in choosing the appropriate drug for treating mastitis. This should be done keeping in view the emergence of multi drug resistant strains.

## Authors’ Contributions

AMB, JSS and RS designed the study. IAD and MYD helped in execution of the work. TH and MM drafted and revised the manuscript. All authors have read the manuscript and approved it.
